# Alterations to the Frequency and Function of Peripheral Blood Monocytes and Associations with Chronic Disease in the Advanced-Age, Frail Elderly

**DOI:** 10.1371/journal.pone.0104522

**Published:** 2014-08-08

**Authors:** Chris P. Verschoor, Jennie Johnstone, Jamie Millar, Robin Parsons, Alina Lelic, Mark Loeb, Jonathan L. Bramson, Dawn M. E. Bowdish

**Affiliations:** 1 Department of Pathology and Molecular Medicine, McMaster University, Hamilton, Ontario, Canada; 2 Department of Clinical Epidemiology and Biostatistics, McMaster University, Hamilton, Ontario, Canada; 3 Department of Medicine, McMaster University, Hamilton, Ontario, Canada; 4 Institute for Infectious Diseases Research, McMaster University, Hamilton, Ontario, Canada; University of Lyon, France

## Abstract

**Background:**

Circulating myeloid cells are important mediators of the inflammatory response, acting as a major source of resident tissue antigen presenting cells and serum cytokines. They represent a number of distinct subpopulations whose functional capacity and relative concentrations are known to change with age. Little is known of these changes in the very old and physically frail, a rapidly increasing proportion of the North American population.

**Design:**

In the following study the frequency and receptor expression of blood monocytes and dendritic cells (DCs) were characterized in a sample of advanced-age, frail elderly (81–100 yrs), and compared against that of adults (19–59 yrs), and community-dwelling seniors (61–76 yrs). Cytokine responses following TLR stimulation were also investigated, as well as associations between immunophenotyping parameters and chronic diseases.

**Results:**

The advanced-age, frail elderly had significantly fewer CD14(++) and CD14(+)CD16(+), but not CD14(++)CD16(+) monocytes, fewer plasmacytoid and myeloid DCs, and a lower frequency of monocytes expressing the chemokine receptors CCR2 and CX_3_CR1. At baseline and following stimulation with TLR-2 and -4 agonists, monocytes from the advanced-age, frail elderly produced more TNF than adults, although the overall induction was significantly lower. Finally, monocyte subset frequency and CX_3_CR1 expression was positively associated with dementia, while negatively associated with anemia and diabetes in the advanced-age, frail elderly.

**Conclusions:**

These data demonstrate that blood monocyte frequency and phenotype are altered in the advanced-age, frail elderly and that these changes correlate with certain chronic diseases. Whether these changes contribute to or are caused by these conditions warrants further investigation.

## Introduction

Age-related changes in circulating immune cell composition and levels of circulating pro-inflammatory cytokines have been associated with longevity [1], frailty [2] and age-related diseases such as Alzheimer's and Parkinson's disease [3], and rheumatoid arthritis [4]. Although the original description of the “immune risk phenotype” (a constellation of immunological markers that is predictive of survival in the aged) consisted primarily of T cell markers and levels of circulating pro-inflammatory cytokines [5], recent studies have begun to investigate age-related changes in myeloid cells such as monocytes and dendritic cells [6]–[11]. In the peripheral blood, monocytes can be subdivided into the classical (CD14^++^HLA-DR^+^), intermediate (CD14^++^CD16^+^HLA-DR^+^) and non-classical (CD14^+/dim^CD16^+^HLA-DR^+^) subsets, and dendritic cells can be subdivided into myeloid CD1c^+^HLA-DR^+^ or CD141^++^HLA-DR^+^ subsets and the plasmacytoid (pDCs, CD303^+^HLA-DR^+^ or CD123^++^HLA-DR^+^) subset [12]. For individuals that are particularly susceptible to developing infectious or chronic disease, such as the advanced-age, frail elderly, alterations to these cellular populations may be a sensitive bio-marker in determining their level of risk. These markers could include the frequency of a given cellular subset in the circulation, the expression of receptors that are critical for the migration to tissues via chemokine gradients or the innate response to pathogens, or potentially the *ex vivo* response to an exogenous stimuli.

In the following study, we characterized the frequency of blood monocytes and DCs, as well as their expression of the innate signalling receptors toll-like receptor (TLR) -2 and -4, and the chemokine receptors CCR2 and CX_3_CR1. Furthermore, we sought to test the hypothesis that monocytes from the advanced-age, frail elderly are immunosenescent, and therefore are likely to be less responsive to innate ligands for TLR-2 (Pam3CSK4) and -4 (lipopolysaccharide, LPS), compared to adults. To investigate whether monocyte and DC frequency and phenotype associate with chronic diseases common to the very old, we recruited a second, larger cohort of the advanced-age, frail elderly.

## Methods

### Participants

Young and middle-aged adults (19–59 years old, median = 34, n = 35 (42% female)) and community-dwelling seniors (61–76 years old, median = 69, n = 45 (67% female)) were recruited from Hamilton, Ontario between January and May in 2012. The advanced-age, frail elderly (defined as having a score of at least 4 on the Clinical Frailty Scale [13]) were recruited from five local nursing homes in 2010 and 2012. Participants were excluded if they were currently on immunosuppressive medication and pre-existing diseases were established by review of each participant's medical chart (Table 1). Participants recruited between January and May in 2012 (81–100 years old, median = 89, n = 49, 88% female) were compared against adults and community-dwelling seniors with regards to monocyte and DC frequency and phenotype, while participants recruited between September and December in 2010 (68–99 years old, median = 88, n = 136, 85% female) were examined for associations between those immunophenotyping variables and pre-existing diseases. The latter, second cohort was deemed necessary in order to have sufficient statistical power to perform the desired association tests. For all participants venous blood was collected from all donors by sodium heparin vacutainer (BD Biosciences, NJ, USA). Written informed consent was obtained from all participants or their legally appointed guardian in the event they were not competent to provide consent themselves. These studies and consent procedures and documents were approved by the McMaster Research Ethics Board (#13-05-14).

**Table 1:** Distribution of advanced-age, frail elderly with regards to disease.

	Disease positive			Disease Negative		
	n	Mean Age	M:F	n	Mean Age	M:F
**Anemia**	20	85.5	3∶17	116	86.8	18∶98
**Arrhythmia**	31	86.5	6∶25	105	86.6	15∶90
**Asthma**	11	85.9	0∶11	125	86.7	21∶104
**Coronary artery disease**	37	88.4	8∶29	99	85.9	13∶86
**Congestive heart failure**	16	87.6	2∶14	120	86.5	19∶101
**Chronic obstructive pulmonary disease**	13	86.7	2∶11	123	86.6	19∶104
**Stroke**	17	88.2	3∶14	119	86.4	18∶101
**Dementia**	66	87.0	11∶55	70	86.2	10∶60
**Diabetes mellitus**	32	84.7	6∶26	104	87.2	15∶89

M:F, Male:Female.

### Immunophenotyping procedure

**Figure 1:**
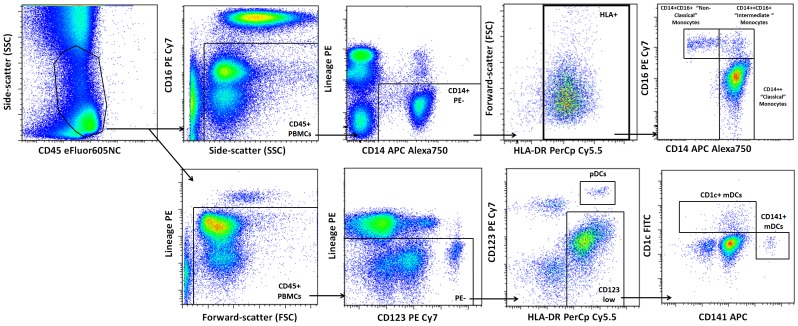
Summary of the gating strategy to define blood monocyte (upper panel) and dendritic cell (lower panel) subsets. Monocytes were defined as CD45 and HLA-DR expressing and lineage (CD2, CD3, CD15, CD19, CD56, NKp46) negative. Dendritic cell subsets were defined as CD45 and HLA-DR expressing, lineage (CD3, CD15, CD19, CD56) negative, and CD123 bright plasmacytoid dendritic cells (pDCs) or CD123 low, and CD1c or CD141 expressing myeloid dendritic cells (mDCs).

Antibody staining was performed as described previously [14]. For the comparison of young adults, community-dwelling seniors and advanced-age, frail elderly, fluorochrome conjugated antibodies included: CD2-PE, CD3-PE, CD16-PE, CD19-PE, CD56-PE, NKp46-PE, CCR2-Alexa647 (BD Biosciences, NJ, USA); CD15-PE, CD1c-FITC, CD141-APC (Miltenyi Biotec, CA, USA); CD14-APC-Alexa750 (Invitrogen, ON, CAN); CX_3_CR1-FITC (Biolegend, CA, USA); CD16-PE-Cy7, HLADR-PerCp-Cy5.5, CD45-eFluor605NC, CD123-PE-Cy7, TLR-4-Alexa700, TLR-2-eFluor450 (eBioscience, CA, USA). For monocyte staining, lineage cells were defined as CD2, CD3, CD15, CD19, CD56 and NKp46 positive, and CD16 thresholds were defined using a fluorescent-minus-one (FMO) with isotype control (Figure 1). For DC staining, lineage cells were defined as CD3, CD15, CD16, CD19 and CD56 positive (Figure 1). Thresholds to determine percentage of cells expressing CCR2, CX_3_CR1, TLR-2 and TLR-4 were calculated using an FMO with isotype control or negative staining population where appropriate. The frequency of monocyte and DC subsets is presented as per µl of whole blood (calculated using CountBright absolute counting beads) as well as the percentage of CD45 expressing PBMCs. Proportions of monocyte and DC subsets were defined as the percentage of CD45 expressing PBMCs. All analyses were performed in FlowJo 7.6.4 (Treestar, OR, USA).

### Intracellular cytokine staining

Intracellular cytokine staining was performed on cryopreserved PBMCs of donors randomly selected from each age group (young adults and advanced-age, frail elderly). Briefly, 10^6^ cells (4×10^6^/ml) in X-VIVO 10 media (Lonza, Basel, CH) supplemented with 10% human AB serum (Lonza, Basel, CH) were treated with PBS (mock), 50 ng/ml LPS (Sigma, MO, USA), or 500 ng/ml Pam3CSK4 (Invivogen, CA, USA), and 1x Protein Transport Inhibitor (eBioscience, CA, USA) for 6 hours at 37°C/5% CO_2_. Surface staining was performed for 30 min at room temperature with the conjugated antibodies CD14-Pacific Blue (Biolegend, CA, USA), CD16 PE-Cy7, HLA-DR-PerCp Cy5.5 (eBioscience, CA, USA) and CD3-AmCyan (BD Bioscience, ON,CA), and fixed with 1x Fix/lyse buffer (eBioscience, CA, USA) for 10 min. Cells were permeabilized for 30 min with 1x Permeabilization Buffer (eBioscience, CA, USA) at room temperature, and stained with the conjugated antibodies TNF-Alexa Fluor 700, IL-1β-PE, IL-8-APC, and IL-6-FITC (eBioscience, CA, USA) for 30 min at room temperature. Cells were fixed with 2% paraformaldehyde, centrifuged and resuspended in FacsWash prior to analysis. Monocytes were defined as high front scatter (FSC)/Side scatter (SSC), and expressing CD14 and/or CD16 and HLA-DR, but not CD3. Flow cytometry and analysis was performed as described above.

### Statistics

All statistical analyses were performed in R 2.11.1 (R Development Core Team, 2011) or Microsoft Excel. For immunophenotyping, differences between age groups were compared using the non-parametric Wilcoxon rank sum test. Experimental-wise significance threshold was determined using the Benjamini-Hochberg procedure for controlling false discovery rate. To determine if donor sex provided substantial bias in our comparisons between age groups, we performed an initial analysis by linear regression on log-transformed values. This indicated that sex only had a significant (experimental-wise p<0.05) effect on the absolute count and proportion of classical monocytes. For associations with disease in the advanced-age, frail elderly, analysis was performed by logistic regression on log-transformed parameters, and adjusted for age. Logarithmic transformation was necessary to approximate normality, and the ratio of males to females was determined to be balanced between cases and controls (Chi-square p>0.05). Comparison of intracellular cytokine production was performed by Student's t-test on log-transformed values.

## Results

For our characterization of peripheral blood monocyte and DC subsets in the advanced-age, frail elderly, we included a cohort of young adults and community-dwelling seniors in order to ascertain whether the cellular frequency and receptor expression levels observed are consistent with alterations that occur over the course of aging, or if they are indeed particular to advanced-age, frail elderly individuals. The absolute frequencies of CD45+ PBMCs were found to significantly decrease with age (mean ± SEM: young adults 2,339±100, seniors 1,893±120 and advanced-age, frail elderly 1,146±107) (Table 2) whereas there was a decrease in the absolute number and percentage of classical monocytes between young adults and the aged (seniors and advanced-age, frail elderly), but no significant decrease between seniors and the advanced-age, frail elderly (Table 2). Consistent with previous studies [8], [10], the ratio of classical to intermediate monocytes is reduced with age and we observe that this reduction is more dramatic in the advanced-age, frail elderly (mean ± SEM: young adults: 25.5±1.8, seniors: 20.5±2.4, advanced-age, frail elderly: 14.4±1.3). As has been previously observed [6], [7], [9], there was a reduction in circulating myeloid (CD1c+ and CD141+) DCs, which we found is further decreased in the advanced-age, frail elderly, while plasmacytoid DCs were significantly reduced in seniors and the advanced-age, frail elderly.

**Table 2:** Immunophenotyping of peripheral blood mononuclear cells (PBMCs) from young adults, community-dwelling seniors, and advanced-age, frail elderly.

		Adults	Seniors	Elderly	Wilcoxon Rank-Sum P-value		
		(19–59 yrs, n = 35)	(61–76 yrs, n = 45)	(81–100 yrs, n = 49)	*AdultxSenior*	*AdultxElderly*	*SeniorxElderly*
**CD45^+^ PBMCs**	Cells/µL	2,339±100	1,893±120	1,146±107	**<0.001**	**<0.001**	<0.001
**CD14^++^ “Classical” monocytes**	Cells/µL	177±11	104±9	98±10	**<0.001**	**<0.001**	-
	Rel. PBMCs (%)	8.36±0.66	5.13±0.42	7.23±0.89	**<0.001**	**0.017**	-
	CCR2^+^ (%)	19.3±3.1	5.1±1.2	8.7±2.3	**<0.001**	**<0.001**	0.170
	CX_3_CR1^+^ (%)	92.9±1.0	90.5±2.1	77.6±3.7	-	**0.005**	**0.002**
	TLR-2^+^ (%)	100.0±0.01	100.0±0.01	100.0±0.01			
	TLR-4^+^ (%)	1.4±0.12	1.4±0.10	1.7±0.09	-	**0.006**	0.061
**CD14^++^CD16^+^ “Intermediate” monocytes**	Cells/µL	7.92±0.70	6.50±0.64	7.77±0.68	0.057	-	0.145
	Rel. PBMCs (%)	0.37±0.03	0.31±0.03	0.58±0.06	-	0.046	**0.001**
	CCR2^+^ (%)	10.2±1.7	2.56±0.40	4.91±1.19	**<0.001**	**<0.001**	-
	CX_3_CR1^+^ (%)	95.7±0.7	93.4±1.6	78.9±3.5	-	**<0.001**	**<0.001**
	TLR-2^+^ (%)	100.0±0.02	99.9±0.03	100.0±0.01	-	-	-
	TLR-4^+^ (%)	2.0±0.43	1.7±0.22	1.6±0.23	-	-	-
**CD14^+^CD16^+^ “Non-classical” monocytes**	Cells/µL	13.9±1.3	13.1±1.3	7.9±0.8	-	**<0.001**	**<0.001**
	Rel. PBMCs (%)	0.64±0.06	0.62±0.05	0.55±0.06	-	0.140	0.160
	CCR2^+^ (%)	1.52±0.24	1.17±0.13	1.05±0.16	-	0.069	0.222
	CX_3_CR1^+^ (%)	99.7±0.1	99.6±0.1	98.5±0.4	-	**0.016**	**0.003**
	TLR-2^+^ (%)	99.3±0.3	99.7±0.1	99.7±0.1	0.100	-	-
	TLR-4^+^ (%)	1.2±0.16	1.2±0.14	1.3±0.15	-	-	-
**Classical/Intermediate ratio**		25.5±1.8	20.5±2.4	14.4±1.3	**0.003**	**<0.001**	**0.014**
**Intermediate/Non-classical ratio**		0.65±0.04	0.55±0.05	1.45±0.19	0.042	**<0.001**	**<0.001**
**CD141^++^ myeloid dendritic cells**	Cells/µL	0.68±0.06	0.67±0.06	0.26±0.03	-	**<0.001**	**<0.001**
	Rel. PBMCs (%)	0.031±0.004	0.038±0.003	0.025±0.002	0.123	0.147	**0.002**
	TLR-2^+^ (%)	26.1±2.1	34.7±3.3	40.2±3.9	0.137	**0.011**	-
	TLR-4^+^ (%)	14.5±1.6	15.7±1.6	17.0±2.3	-	-	-
**CD1c^+^ myeloid dendritic cells**	Cells/µL	11.7±1.2	11.2±0.6	7.3±0.7	0.121	**0.005**	**<0.001**
	Rel. PBMCs (%)	0.52±0.06	0.64±0.04	0.68±0.04	-	-	-
	TLR-2^+^ (%)	90.1±0.8	91.4±0.7	92.4±1.0	-	**0.005**	0.040
	TLR-4^+^ (%)	16.6±0.8	17.6±1.1	19.3±1.2	-	-	-
**CD123^++^ plasmacytoid dendritic cells**	Cells/µL	7.39±0.56	4.03±0.22	2.45±0.31	**<0.001**	**<0.001**	**<0.001**
	Rel. PBMCs (%)	0.32±0.02	0.23±0.01	0.22±0.02	**0.004**	**0.002**	-
	TLR-2^+^ (%)	11.3±1.2	13.5±1.1	10.0±1.0	0.094	-	**0.017**
	TLR-4^+^ (%)	5.2±0.39	5.6±0.55	6.5±0.66	-	-	-

Mean and standard error presented. Only values with comparison-wise (Wilcoxon rank-sum test) significance at p<0.25 shown; Bolded values indicated experimental-wise (Benjamin-Hochberg FDR) significance at p<0.05. Cells/µL, cells per microliter of blood (absolute count); Rel. PBMCs, relative to PBMCs.

In addition to measurements of frequency, the expression of innate pattern recognition receptors TLR-2 and TLR-4 were measured on monocytes and DCs, and the expression of chemokine receptors CX_3_CR1 and CCR2 on monocytes alone (Table 2). It would appear that the percentage of TLR-2 expressing myeloid DCs is increased in the advanced-age, frail elderly, while no differences were observed for monocytes. It should be noted that although the trends regarding TLR-2 expression suggest an increase from young adults, to seniors, to the advanced-age, frail elderly. However, the subtlety in these alterations and degree of variation do not allow us to conclude as such. A subtle, but significant increase in the percentage of TLR-4 expressing classical monocytes was also observed in the advanced-age, frail elderly. There is an age-related reduction in the percentage of monocytes expressing CCR2, but no significant difference between community-dwelling seniors and the advanced-age, frail elderly. For CX_3_CR1 however, a reduction in the percentage of expressing monocytes appears to be limited to the advanced-age, frail elderly.

**Figure 2:**
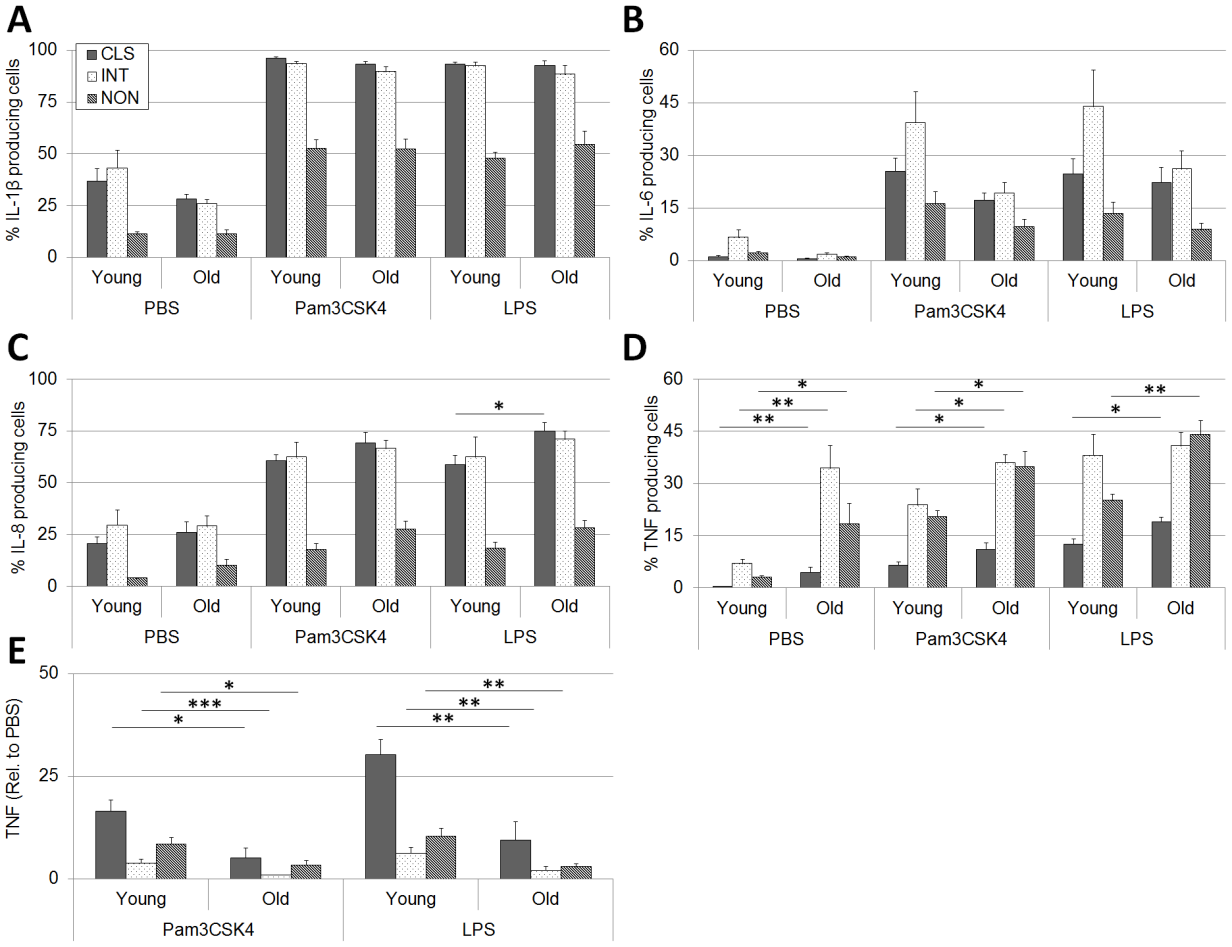
Cytokine production, but not induction, is elevated in monocytes from the advanced-age, frail elderly as compared to young adults. PBMCs were stimulated with mock (PBS), and TLR-2 (Pam3CSK4, Pam) and TLR-4 (LPS) agonists, and the production of A) IL-1β, B) IL-6, C) IL-8 and D) TNF in classical (CLS), intermediate (INT) and non-classical (NON) monocytes was measured by flow cytometry. Relative to mock, the induction of E) TNF was significantly lower in the advanced-age, frail elderly. n = 5–8 per group, per treatment. Comparison-wise p-value, ***p<0.001, **p<0.01, *p<0.05.

Although only subtle differences in the expression of TLR-2 and -4 were observed for monocytes from the advanced-age, frail elderly, we sought to additionally characterize the functional capacity of monocyte subsets from this age group to respond to stimulus via these receptors. Using intracellular cytokine staining, the production of IL-1β, IL-6, IL-8 and TNF by monocytes subsets in response to Pam3CSK4 (TLR-2 agonist) and LPS (TLR-4 agonist) were quantified in PBMCs from the advanced-age, frail elderly and young adults. Consistent with previous literature [11], [15], [16] the relative production of cytokine by monocyte subsets are as follows: IL-1β, Classical  =  Intermediate > Non-classical; IL-6, Intermediate > Classical > Non-classical; IL-8, Classical  =  Intermediate > Non-classical; TNF, Intermediate ≥ Non-classical > Classical (Figure 2A-D). No significant differences between age-groups in the overall production of IL-1β (Figure 2A) or IL-6 (Figure 2B) were observed. Classical monocytes from the advanced-age, frail elderly produced more IL-8 in response to LPS as compared to young adults (Figure 2D), and for all subsets, with exception to intermediate monocytes stimulated with LPS, TNF production was greater in the advanced-age, frail elderly at baseline (PBS mock control) and in response to Pam3CSK4 or LPS (Figure 2D). Interestingly, while the overall production of TNF was greater in the advanced-age, frail elderly, the relative induction of TNF (versus PBS mock control) was significantly lower for all subsets compared to young adults (Figure 2E). No differences between age-groups were observed for the relative induction of IL-1β, IL-6 or IL-8 (data not shown).

**Figure 3:**
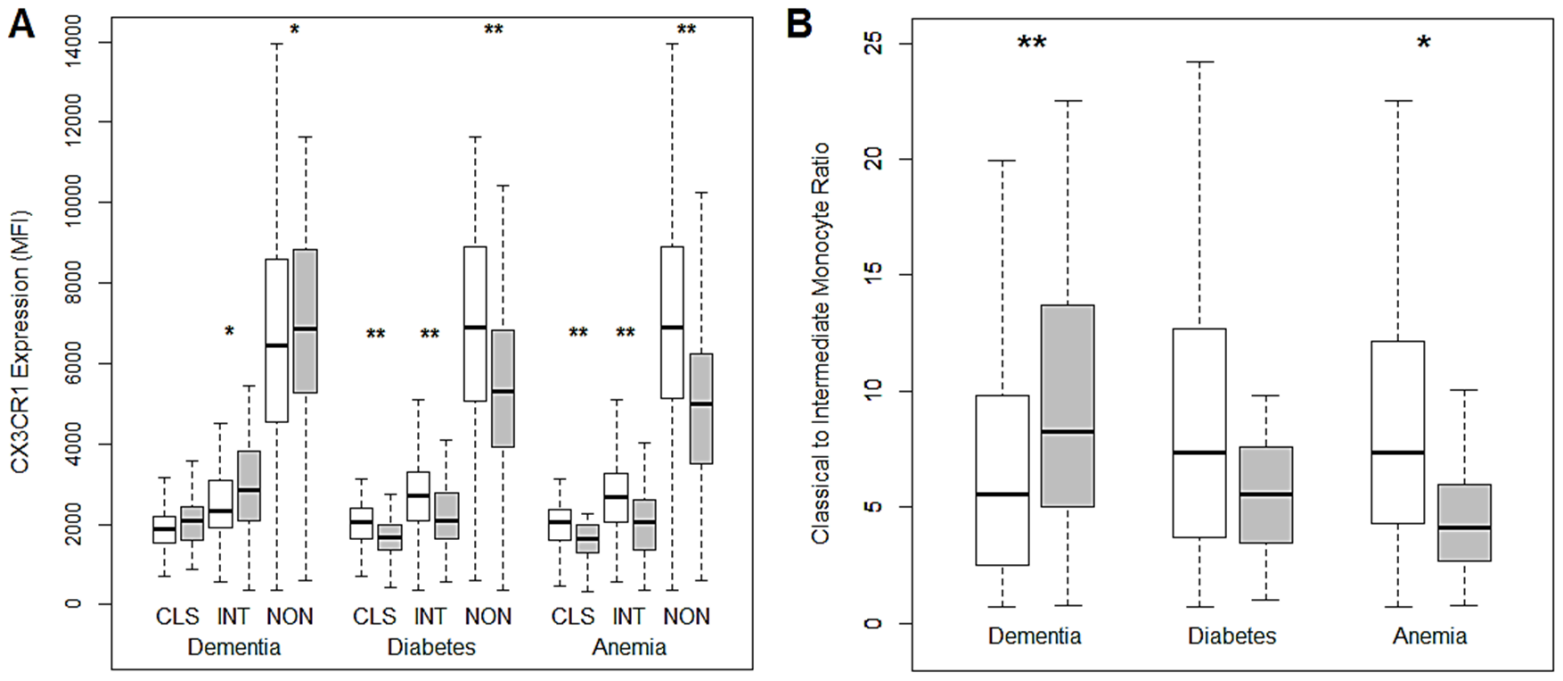
Representation of the differences in A) the expression of CX_3_CR1 on the classical (CLS), intermediate (INT) and non-classical (NON) monocyte subsets and B) the classical to intermediate monocyte ratio, between cases (grey) and controls (white) for dementia, diabetes mellitus and anemia. Comparison-wise p-value, **p<0.01, *p<0.05.

To determine whether the observed alterations to monocyte and DC frequency and monocyte CCR2 and CX_3_CR1 expression in the advanced-age, frail elderly are associated with chronic disease, we analyzed a larger, second cohort of 136 participants (Table 1). This cohort second cohort was deemed necessary in order to have sufficient statistical power to perform the desired association tests. Within the advanced-age, frail elderly cohort we performed logistic regression for each of the monocyte and DC markers in a univariate manner against the presence of chronic obstructive pulmonary disease, congestive heart failure, coronary artery disease, asthma, dementia, cerebral vascular accident, diabetes mellitus, arrhythmia or anemia (Table 3). Other than a positive association between pDC frequency and dementia, no significant associations were observed for the frequencies of blood DCs. Although the senior and advanced-age, frail elderly groups had fewer monocytes expressing CCR2, there was no statistically significant association between monocyte CCR2 expression and disease (data not shown). In contrast, reductions in CX_3_CR1 expression only occurred in the advanced-age, frail elderly and individuals with elevated levels of CX_3_CR1 had a greater likelihood of having dementia, while reduced expression was associated with an increased risk of diabetes and anemia (Figure 3A). In addition there was a significant correlation between monocyte frequency and dementia, diabetes and anemia. The likelihood of having dementia was positively associated with monocytes and the classical to intermediate monocyte ratio, whereas for diabetes and anemia, opposite trends were observed (Figure 3B).

**Table 3:** Associations between blood monocyte and DC markers and disease in the advanced-age, frail elderly.

		Chronic obstructive pulmonary disease	Congestive heart failure	Coronary artery disease	Asthma	Dementia	Cerebral vascular accident	Diabetes mellitus	Arrhythmia	Anemia
**Monocytes**	*CD45%*	2.47 (0.70–9.24)	-	-	-	**2.49 (1.16–5.66)** *	-	0.44 (0.17–1.12)	-	**0.33 (0.10–0.99)**
**Classical**	*CD45%*	2.13 (0.80–6.16)	-	-	-	1.66 (0.95–3.02)	-	**0.52 (0.26–1.03)**	0.60 (0.30–1.15)	**0.40 (0.18–0.88)** *
	*CX_3_CR1 (MFI)*	-	-	1.93 (0.70–6.05)	0.24 (0.06–1.01) *	2.07 (0.83–5.62)	-	**0.22 (0.07–0.62)** **	-	**0.20 (0.06–0.64)** **
**Intermediate**	*CD45%*	-	-	1.55 (0.91–2.69)	-	0.67 (0.41–1.06)	0.52 (0.25–1.05)	-	1.89 (1.08–3.46) *	-
	*CX_3_CR1 (MFI)*	-	-	-	0.24 (0.06–0.91) *	**2.90 (1.24–7.41)** *	-	**0.19 (0.06–0.50)** **	0.43 (0.16–1.08)	**0.18 (0.05–0.52)** **
**Non-classical**	*CD45%*	-	-	-	-	1.41 (0.84–2.40)	-	**0.35 (0.18–0.67)** **	-	0.54 (0.26–1.09)
	*CX_3_CR1 (MFI)*	-	-	1.72 (0.82–4.15)	0.38 (0.14–1.02) *	**2.07 (1.04–4.48)** *	-	**0.32 (0.14–0.67)** **	-	**0.32 (0.13–0.72)** **
**Classical:Intermediate ratio**		1.69 (0.87–3.48)	-	0.76 (0.49–1.17)	-	**1.74 (1.16–2.69)** **	1.68 (0.92–3.23)	0.72 (0.45–1.15)	**0.49 (0.30–0.79)** **	**0.55 (0.31–0.94)** *
**pDC**	*CD45%*	-	-	-	-	**0.56 (0.30–0.98)** *	-	0.51 (0.25–1.00)	-	-
**CD1c+ mDC**	*CD45%*	-	-	-	2.79 (0.71–12.07)	-	-	0.44 (0.18–1.08)	-	-
**CD141+ mDC**	*CD45%*	-	-	-	-	-	-	-	-	0.56 (0.28–1.12)

Odds ratios and 95% confidence intervals presented. Only comparison-wise (logistic regression) significance at p<0.25 shown;

*p<0.05,

**p<0.01. Bolded observations indicate experimental-wise (Benjamini-Hochberg FDR) significance at p<0.15. CD45%, percentage of cells relative to CD45+ PBMCs; MFI, mean fluorescent intensity.

## Discussion

Our results indicate that for many, but not all myeloid cell populations, age-related alterations tend to become more pronounced with advanced-age and frailty. These changes in circulating myeloid cell populations may reflect changes in precursor generation or emigration from the bone marrow. As an example, the recent finding in mice that a reduction of circulating pDCs stimulates myelopoiesis and increases circulating myeloid-derived suppressor cells (MDSCs) [17], whose numbers increase in the advanced-age, frail elderly [14], implies that there may be previously unappreciated feedback mechanisms between circulating DCs and the bone marrow compartment.

In addition to changes in frequency, changes in phenotype and function have been shown to occur with age and it has been proposed that these phenotypic changes may contribute to age-associated chronic disease, especially those with inflammatory etiology. There have been conflicting reports, for example, as to whether monocytes have hypo- or hyper-inflammatory responses to TLR ligands and whether these might be due to changes in TLR expression [18], [19]. While we observed only a slight increase in the percentage of TLR-4 expressing classical monocytes and no changes to the expression of monocyte TLR-2, the production of TNF, and to a lesser extent IL-8, was significantly higher in monocyte subsets from the advanced-age, frail elderly, both at baseline and in response to TLR-2 and -4 stimuli. This is similar to what has been shown in previous reports [8], [11], and supports the theory that constitutive over-production of cytokines by monocyte subsets may predispose elderly individuals to a higher risk of chronic disease.

Another potential contributor to the development of chronic disease in the elderly is the ability of circulating monocytes to migrate to the tissues. Both CCR2 and CX_3_CR1, receptors for the chemokines MCP-1 (CCL2) and fractalkine (CX_3_CL1), are potently involved in the migration and recruitment of monocytes in the host. Monocyte recruitment via chemokine receptors has been linked to the development of inflammatory diseases such as atherosclerosis and cancer [20], [21], and an increased expression of the monocyte/macrophage chemoattractant CX_3_CL1 has been observed in cardiovascular disease and Alzheimer's disease [22], [23]. Although correlations of increased CX_3_CR1 expression and dementia have not been previously demonstrated in humans, our observations are consistent with mouse models of Alzheimer's disease in which either a loss of CX_3_CR1 gene expression [24] or a reduction in signalling through CX_3_CR1 [25] results in an improved outcome. Little is known regarding monocyte phenotype or CX_3_CR1 expression and diabetes, although it has been shown that monocytes display an activated phenotype in diabetics [26], [27] and the production of CX_3_CL1, likely by adipocytes, is found at higher levels in diabetics [28]. Whether monocytes expressing lower levels of CX_3_CR1 contribute to insulin resistance and diabetes in the frail elderly is not known. We also observed associations of decreased monocyte numbers and CX_3_CR1 expression with anemia. Chronic inflammation anemia is extremely common in the frail elderly [29] and is associated with elevated levels of inflammatory cytokines, especially IL-6 [30]. Since monocytes and erythrocytes share a common progenitor in the bone marrow, this association may be due to a common mechanism of suppressed myelopoeisis due to the aging or the immune status of the host.

In summary, changes in monocyte frequency, phenotype and function occur in the advanced-age, frail elderly and correlate with chronic disease. However, we do not know if these changes predispose individuals to age-related diseases or whether the overall immune status associated with many of these conditions is what ultimately impacts monocyte development and function. Future longitudinal studies will need to be performed to dissect the cause and effect of these changes as individuals approach advanced-age. Changes in circulating monocyte frequency and phenotype may be robust markers of immune risk in the aged.

## References

[1] Larbi A, Franceschi C, Mazzatti D, Solana R, Wikby A, et al (2008) Aging of the immune system as a prognostic factor for human longevity. Physiology (Bethesda) 23: 64–74 23/2/64 [pii];10.1152/physiol.00040.2007 [doi]. 18400689 10.1152/physiol.00040.2007

[2] van den Biggelaar AH, Huizinga TW, de Craen AJ, Gussekloo J, Heijmans BT, et al (2004) Impaired innate immunity predicts frailty in old age. The Leiden 85-plus study. Exp Gerontol 39: 1407–1414 S0531-5565(04)00213-X [pii];10.1016/j.exger.2004.06.009 [doi]. 15489064 10.1016/j.exger.2004.06.009

[3] Rosenkranz D, Weyer S, Tolosa E, Gaenslen A, Berg D, et al (2007) Higher frequency of regulatory T cells in the elderly and increased suppressive activity in neurodegeneration. J Neuroimmunol 188: 117–127 S0165-5728(07)00174-9 [pii];10.1016/j.jneuroim.2007.05.011 [doi]. 17582512 10.1016/j.jneuroim.2007.05.011

[4] Korkosz M, Bukowska-Strakova K, Sadis S, Grodzicki T, Siedlar M (2012) Monoclonal antibodies against macrophage colony-stimulating factor diminish the number of circulating intermediate and nonclassical (CD14(++)CD16(+)/CD14(+)CD16(++)) monocytes in rheumatoid arthritis patient. Blood 119: 5329–5330 119/22/5329 [pii];10.1182/blood-2012-02-412551 [doi]. 22653956 10.1182/blood-2012-02-412551

[5] Wikby A, Maxson P, Olsson J, Johansson B, Ferguson FG (1998) Changes in CD8 and CD4 lymphocyte subsets, T cell proliferation responses and non-survival in the very old: the Swedish longitudinal OCTO-immune study. Mech Ageing Dev 102: 187–198. 9720651 10.1016/s0047-6374(97)00151-6

[6] Della BS, Bierti L, Presicce P, Arienti R, Valenti M, et al (2007) Peripheral blood dendritic cells and monocytes are differently regulated in the elderly. Clin Immunol 122: 220–228 S1521-6616(06)00902-8 [pii];10.1016/j.clim.2006.09.012 [doi]. 17101294 10.1016/j.clim.2006.09.012

[7] Jing Y, Shaheen E, Drake RR, Chen N, Gravenstein S, et al (2009) Aging is associated with a numerical and functional decline in plasmacytoid dendritic cells, whereas myeloid dendritic cells are relatively unaltered in human peripheral blood. Hum Immunol 70: 777–784 S0198-8859(09)00171-2 [pii];10.1016/j.humimm.2009.07.005 [doi]. 19596035 10.1016/j.humimm.2009.07.005 PMC5718338

[8] Nyugen J, Agrawal S, Gollapudi S, Gupta S (2010) Impaired functions of peripheral blood monocyte subpopulations in aged humans. J Clin Immunol 30: 806–813 10.1007/s10875-010-9448-8 [doi]. 20703784 10.1007/s10875-010-9448-8 PMC2970801

[9] Perez-Cabezas B, Naranjo-Gomez M, Fernandez MA, Grifols JR, Pujol-Borrell R, et al (2007) Reduced numbers of plasmacytoid dendritic cells in aged blood donors. Exp Gerontol 42: 1033–1038 S0531-5565(07)00130-1 [pii];10.1016/j.exger.2007.05.010 [doi]. 17606348 10.1016/j.exger.2007.05.010

[10] Seidler S, Zimmermann HW, Bartneck M, Trautwein C, Tacke F (2010) Age-dependent alterations of monocyte subsets and monocyte-related chemokine pathways in healthy adults. BMC Immunol 11: 30 1471-2172-11-30 [pii];10.1186/1471-2172-11-30 [doi]. 20565954 10.1186/1471-2172-11-30 PMC2910032

[11] Hearps AC, Martin GE, Angelovich TA, Cheng WJ, Maisa A, et al (2012) Aging is associated with chronic innate immune activation and dysregulation of monocyte phenotype and function. Aging Cell 11: 867–875 10.1111/j.1474-9726.2012.00851.x [doi]. 22708967 10.1111/j.1474-9726.2012.00851.x

[12] Ziegler-Heitbrock L, Ancuta P, Crowe S, Dalod M, Grau V, et al (2010) Nomenclature of monocytes and dendritic cells in blood. Blood 116: e74–e80 blood-2010-02-258558 [pii];10.1182/blood-2010-02-258558 [doi]. 20628149 10.1182/blood-2010-02-258558

[13] Rockwood K, Abeysundera MJ, Mitnitski A (2007) How should we grade frailty in nursing home patients? J Am Med Dir Assoc 8: 595–603 S1525-8610(07)00351-9 [pii];10.1016/j.jamda.2007.07.012 [doi]. 17998116 10.1016/j.jamda.2007.07.012

[14] Verschoor CP, Johnstone J, Millar J, Dorrington MG, Habibagahi M, et al.. (2013) Blood CD33(+)HLA-DR(-) myeloid-derived suppressor cells are increased with age and a history of cancer. J Leukoc Biol. jlb.0912461 [pii];10.1189/jlb.0912461 [doi]. 10.1189/jlb.0912461 PMC3701116 23341539

[15] Cros J, Cagnard N, Woollard K, Patey N, Zhang SY, et al (2010) Human CD14dim monocytes patrol and sense nucleic acids and viruses via TLR7 and TLR8 receptors. Immunity 33: 375–386 S1074-7613(10)00317-1 [pii];10.1016/j.immuni.2010.08.012 [doi]. 20832340 10.1016/j.immuni.2010.08.012 PMC3063338

[16] Sanchez-Torres C, Garcia-Romo GS, Cornejo-Cortes MA, Rivas-Carvalho A, Sanchez-Schmitz G (2001) CD16+ and CD16- human blood monocyte subsets differentiate in vitro to dendritic cells with different abilities to stimulate CD4+ T cells. Int Immunol 13: 1571–1581. 11717198 10.1093/intimm/13.12.1571

[17] Ioannou M, Alissafi T, Boon L, Boumpas D, Verginis P (2013) In Vivo Ablation of Plasmacytoid Dendritic Cells Inhibits Autoimmunity through Expansion of Myeloid-Derived Suppressor Cells. J Immunol 190: 2631–2640 jimmunol.1201897 [pii];10.4049/jimmunol.1201897 [doi]. 23382560 10.4049/jimmunol.1201897 PMC3586977

[18] Balistreri CR, Colonna-Romano G, Lio D, Candore G, Caruso C (2009) TLR4 polymorphisms and ageing: implications for the pathophysiology of age-related diseases. J Clin Immunol 29: 406–415 10.1007/s10875-009-9297-5 [doi]. 19459036 10.1007/s10875-009-9297-5

[19] Renshaw M, Rockwell J, Engleman C, Gewirtz A, Katz J, et al (2002) Cutting edge: impaired Toll-like receptor expression and function in aging. J Immunol 169: 4697–4701. 12391175 10.4049/jimmunol.169.9.4697

[20] Qian BZ, Li J, Zhang H, Kitamura T, Zhang J, et al (2011) CCL2 recruits inflammatory monocytes to facilitate breast-tumour metastasis. Nature 475: 222–225 nature10138 [pii];10.1038/nature10138 [doi]. 21654748 10.1038/nature10138 PMC3208506

[21] Swirski FK, Nahrendorf M (2013) Leukocyte behavior in atherosclerosis, myocardial infarction, and heart failure. Science 339: 161–166 339/6116/161 [pii];10.1126/science.1230719 [doi]. 23307733 10.1126/science.1230719 PMC3891792

[22] Kim TS, Lim HK, Lee JY, Kim DJ, Park S, et al (2008) Changes in the levels of plasma soluble fractalkine in patients with mild cognitive impairment and Alzheimer's disease. Neurosci Lett 436: 196–200 S0304-3940(08)00319-4 [pii];10.1016/j.neulet.2008.03.019 [doi]. 18378084 10.1016/j.neulet.2008.03.019

[23] Wong BW, Wong D, McManus BM (2002) Characterization of fractalkine (CX3CL1) and CX3CR1 in human coronary arteries with native atherosclerosis, diabetes mellitus, and transplant vascular disease. Cardiovasc Pathol 11: 332–338. S1054880702001114 [pii]. 10.1016/s1054-8807(02)00111-4 12459434

[24] Fuhrmann M, Bittner T, Jung CK, Burgold S, Page RM, et al (2010) Microglial Cx3cr1 knockout prevents neuron loss in a mouse model of Alzheimer's disease. Nat Neurosci 13: 411–413 nn.2511 [pii];10.1038/nn.2511 [doi]. 20305648 10.1038/nn.2511 PMC4072212

[25] Lee S, Varvel NH, Konerth ME, Xu G, Cardona AE, et al (2010) CX3CR1 deficiency alters microglial activation and reduces beta-amyloid deposition in two Alzheimer's disease mouse models. Am J Pathol 177: 2549–2562 S0002-9440(10)60305-7 [pii];10.2353/ajpath.2010.100265 [doi]. 20864679 10.2353/ajpath.2010.100265 PMC2966811

[26] Cipolletta C, Ryan KE, Hanna EV, Trimble ER (2005) Activation of peripheral blood CD14+ monocytes occurs in diabetes. Diabetes 54: 2779–2786. 54/9/2779 [pii]. 10.2337/diabetes.54.9.2779 16123369

[27] Min D, Brooks B, Wong J, Salomon R, Bao W, et al (2012) Alterations in monocyte CD16 in association with diabetes complications. Mediators Inflamm 2012: 649083 10.1155/2012/649083 [doi]. 23316106 10.1155/2012/649083 PMC3536440

[28] Shah R, Hinkle CC, Ferguson JF, Mehta NN, Li M, et al (2011) Fractalkine is a novel human adipochemokine associated with type 2 diabetes. Diabetes 60: 1512–1518 60/5/1512 [pii];10.2337/db10-0956 [doi]. 21525510 10.2337/db10-0956 PMC3292325

[29] Artz AS, Fergusson D, Drinka PJ, Gerald M, Bidenbender R, et al.. (2004) Mechanisms of unexplained anemia in the nursing home. J Am Geriatr Soc 52: 423–427. 52116 [pii]. 10.1111/j.1532-5415.2004.52116.x 14962159

[30] Ershler WB (2003) Biological interactions of aging and anemia: a focus on cytokines. J Am Geriatr Soc 51: S18–S21. jgs5102 [pii]. 10.1046/j.1532-5415.51.3s.2.x 12588568

